# Blood neurofilament light chain and S100B as biomarkers of neurological involvement and functional prognosis in COVID-19: a multicenter study

**DOI:** 10.1007/s10072-024-07964-0

**Published:** 2025-01-08

**Authors:** Francesca Bisulli, Lorenzo Muccioli, Lisa Taruffi, Roberta Bedin, Silvia Felici, Corrado Zenesini, Flavia Baccari, Mauro Gentile, Niccolò Orlandi, Simone Rossi, Marianna Nicodemo, Fabio d’Achille, Pierluigi Viale, Stefania Zaccaroni, Raffaele Lodi, Rocco Liguori, Andrea Zini, Maria Guarino, Pietro Cortelli, Tiziana Lazzarotto, Damir Janigro, Stefano Meletti

**Affiliations:** 1 IRCCS Istituto Delle Scienze Neurologiche Di Bologna, Ospedale Bellaria, Via Altura 3, 40139 Bologna, Italy; 2 Department of Biomedical and Neuromotor Sciences, University of Bologna, Bologna, Italy; 3 Department of Biomedical, Metabolic, and Neural Sciences, University of Modena and Reggio Emilia, Modena, Italy; 4 Neurophysiology Unit and Epilepsy Centre, Neuroscience Department, AOU Modena, Modena, Italy; 5 Dipartimento Interaziendale Ad Attività Integrata Medicina Di Laboratorio E Anatomia Patologica (DIIMLAP), AUSL Modena, Modena, Italy; 6 IRCCS Azienda Ospedaliero Universitaria Di Bologna, Bologna, Italy; 7 Department of Medical and Surgical Sciences, University of Bologna, Bologna, Italy; 8 Azienda USL Di Bologna, Bologna, Italy; 9 FloTBI Inc., Cleveland, OH USA; 10 Department of Physiology and Biophysics, Case Western Reserve University, Cleveland, OH USA

**Keywords:** Neuro-COVID, SARS-CoV-2, Biomarker, Prognosis, NfL, S100B

## Abstract

**Background and aim:**

COVID-19 is associated with neurological complications, termed neuro-COVID, affecting patient outcomes. We aimed to evaluate the association between serum neurofilament light chain (NfL) and S100B biomarkers with the presence of neurological manifestations and functional prognosis in COVID-19 patients.

**Methods:**

A multicenter prospective cohort study was conducted in three hospitals in the Emilia-Romagna region, Italy, from March 2020 to April 2022. Hospitalized patients with PCR-confirmed COVID-19 were enrolled. Serum S100B and NfL levels were measured in the acute or subacute phase after admission. Diagnostic accuracy was assessed using receiver operating characteristic (ROC) analyses. Statistical analyses were performed to evaluate the association between biomarkers, clinical/laboratory variables, and prognosis, specifically focusing on worsening of the modified Rankin Scale (mRS) from admission to discharge.

**Results:**

A total of 279 patients (153 males, median age 76.7 years) were included. Among them, 69 (24.7%) developed neuro-COVID. Serum NfL levels were significantly higher in the neuro-COVID group (median 110 vs 68.3; *p* = 0.035) and correlated with severe encephalopathy and extracranial neurologic manifestations. The ROC analysis showed low accuracy in the discrimination between the two groups for both NfL and S100B. Key predictors of worsening mRS included mechanical ventilation (OR = 9.56, 95% CI = 1.67–54.75; *p* = 0.011), severe encephalopathy (OR = 5.10, 95% CI = 1.58–16.19; *p* = 0.006), and elevated S100B levels (OR = 2.62, 95% CI = 1.10–6.46; *p* = 0.037).

**Conclusions:**

Serum NfL and S100B biomarkers were not accurate in discriminating neuro-COVID patients, however NfL levels were associated with severe and extracranial neuro-COVID, while S100B with functional outcomes, potentially informing clinical management.

## Introduction

The global outbreak of the coronavirus disease 2019 (COVID-19) has led to an unprecedented health crisis, challenging healthcare systems worldwide. As researchers and healthcare professionals strive to better understand the dynamics of the virus and its varied clinical manifestations, the identification and utilization of biomarkers have emerged as promising tools in both prognosis and diagnosis [1]. Biomarkers, encompassing a diverse range of measurable indicators, offer valuable insights into the pathophysiology of COVID-19, aiding in risk stratification and disease monitoring [2, 3].

Since the beginning of the pandemic, it was evident that COVID-19 does not only affect the respiratory system, since other organs are targeted by the virus or sensitive to adaptive changes following infection. In particular, virus-related neurological manifestations have been reported (neuro-COVID). These can be divided into acute [4], occurring during the active infection by the virus, or delayed after the viral infection ceases (long COVID [5, 6]).

The features of neuro-COVID have been described in several publications (reviewed in [7, 8]). However, most available data were gathered by varied approaches (single centers, small and selected samples, case reports) and in heterogeneous populations. Furthermore, published studies often adopted different diagnostic criteria and assessment of the course of the disease [9]. Thus, a systematic literature review may not allow for a reproducible strategy to assess disease severity and neuro-COVID risk.

Neurological complications of COVID-19 include encephalopathies, stroke, seizures and status epilepticus, myelitis, cranial and peripheral neuropathies, and myopathies [7, 8, 10]. These are evident even in the absence of detectable virus in cerebrospinal fluid (CSF) [7, 10, 11]. Most studies found elevated albumin in CSF, an indicator of blood–brain barrier (BBB) disruption [12–14]; in vitro models also revealed that the BBB can be disrupted by the virus [15]. Finally, the peripheral BBB indicator S100B [16, 17] was found elevated in the blood of COVID-19 patients [12], and correlated with disease severity [18]. Taken together, these data suggest that monitoring BBB function in COVID-19 patients may be helpful in aggressively pursuing neurological complications in a timely manner.

However, the data using S100B to predict COVID-19 or to classify disease severity are contradictory. In an early study, a positive correlation between S100B and disease severity was shown [18], but others found no correlation [19]. An additional peripheral biomarker has been proposed as a prognostic tool in neuro-COVID, namely the neurofilament light chain (NfL) [20]. NfL was found elevated in neuro-COVID in a few studies [21, 22] while others found no correlation [19]. The inconsistent evidence surrounding the role of NfL may stem from the possibility that subtle neuroaxonal injury can occur in asymptomatic and pauci-symptomatic COVID-19 patients, and that various factors might influence NfL levels, making this biomarker non-specific. [23] Similar results were obtained with the glial fibrillar acid protein (GFAP), a glia-derived biomarker [19, 21, 24].

A recurring motif across all biomarker studies centered on establishing correlations between biomarker levels and either disease severity or the manifestation of neuro-COVID. However, due to the aforementioned reasons, the results were largely inconclusive.

Herein, we aimed to evaluate the association between serum NfL and S100B with the presence of neurological manifestations and functional prognosis in COVID-19 patients, while also considering comparison of biomarker levels with other clinical and laboratory variables.

## Methods

The STARD (Standards for Reporting Diagnostic accuracy studies) [25] and STROBE (Strengthening the Reporting of Observational Studies in Epidemiology) [26] guidelines were followed.

### Study design, setting and population

A multicenter prospective cohort study design was applied. Consecutive patients admitted to three hospitals in the Emilia-Romagna region in Italy (S.Orsola-Malpighi and Maggiore Hospital in Bologna, Baggiovara Hospital in Modena) with PCR-proven COVID-19, were enrolled between March 2020 and April 2022. Written consent was gained from either patients themselves, or from their legal representatives where they lacked the capacity to consent. This study was approved by the local ethics committee (925–2021-OSS-AUSLBO-21182).

### Variables

Demographic, clinical and laboratory informations were collected by the clinical team at the time of sample collection and reported in the medical records, which have been consulted for the obtainment of pertinent data for the present study.

Clinical variables included: comorbidities (neurologic and respiratory disorders, hypertension, ischemic heart disease, chronic kidney disease, obesity, diabetes, immunosuppression), smoke, fever, treatments (amines, steroids, tocilizumab), and disease course, comprensive of modified Rankin score (mRs) at admission and discharge, were also recorded. COVID-19 severity was defined based on the National Institute of Health guidelines [27] into four classes: no need for oxygen therapy, low-flow oxygen therapy, high-flow oxygen therapy or non-invasive ventilation and mechanical ventilation (MV).

Laboratory variables included: complete blood count, electrolytes, liver, renal and thyroid function tests, coagulation, and inflammatory markers as Interleukin 6 (IL-6) and C-reactive Protein (CRP).

### Index tests – S100B and NfL biomarkers

Blood samples were collected at a single time point in the acute or subacute phase after admission and processed within two hours. Serum was aliquoted into cryovials, labeled with pseudo-anonymized identifiers, and stored at − 80 °C until analysis. Analyses were performed at the Neuroimmunology lab of the OCB Modena Academic Hospital. S100B and NfL were assessed in serum samples and were used as index test in the diagnostic analysis.

### Reference standard – neuro-COVID diagnosis

Patients with a newly diagnosed neurological disorder of any type, developed during the prodromal, acute respiratory, or recovery phase of COVID-19 infection during hospitalization, were eligible, yet they were included in the study solely after a comprehensive evaluation by neurology specialists. Patients with isolated symptoms (such as headache or dizziness) with no concomitant alterations at the neurological examination or congruent findings at the neurophysiological and neuroimaging investigations were excluded. Neurological consultations were conducted in the neurology departments, stroke units, Neuro-COVID wards, and neuro-intensive care units (ICUs). Whenever neuro-COVID symptoms were confirmed, the patient was offered the opportunity to participate in the study and classified as neuro-COVID, in contrast to the “control” group of patients without neurological manifestations (COVID-w/o Neuro). Neuro-COVID patients were further classified as follows: extra-cranial neuro-COVID (e.g., neuropathies, radicoloneuritis, myelitis), mild encephalopathy (e.g., confusion, agitation, ideomotor slowing, epileptic seizure) and severe encephalopathy (e.g., delirium, status epilepticus, coma, and stroke).

### Prognostic analysis

The outcome for the prognostic evaluation was the difference between mRs at discharge and mRS at hospital admission: at least one-unit increase indicated the worsening of the disease (dichotomous outcome: mRS worsening vs. no worsening). The association with the outcome was evaluated for demographic, clinical and laboratory variables.

### Statistical analysis

Continuous descriptive variables were presented using median and interquartile range (IQR). The Shapiro–Wilk test was used to evaluate the normal data distribution. Categorical variables were presented using absolute numbers (n) and percentages (%). Two-group comparisons were assessed using Mann–Whitney U-test for continuous variables and with the Chi-square test for categorical variables.

In the diagnostic analysis receiver operating characteristic (ROC) analyses with relative 95% Confidence Intervals (95% CI) were performed to evaluate the diagnostic accuracy of NfL and S100B biomarkers between Neuro-COVID and COVID patients without neurological symptoms (COVID-w/o Neuro). We calculated sensitivity and specificity according to the optimal cut-off using the Youden Index method.

Spearman’s Rho correlation for continuous variables, and Mann–Whitney U-test for categorical variables, were used to evaluate the variables associated with the two biomarkers. The results were presented with Rho coefficients.

In the cross-sectional analysis (time of sample collection) univariable and multivariable linear regression models were used to evaluate the association between variables and biomarkers (dependent variables). The biomarker levels were not normally distributed and were transformed into natural logarithms. The results were presented with Beta coefficients (β) and 95% CI.

In the prognostic analysis univariable and multivariable logistic regression models were used to evaluate the association between the variables and worsening of disease (at least one-unit increase of mRs scale from hospital admission to discharge—dependent variable). The results were presented with Odds Ratio (OR) and 95% CI.

We conducted univariable analyses for all variables outlined in the methods section. Variables that were associated with the outcome in the univariable analysis were subsequently included in multivariable regression models (both linear and logistic), using a stepwise approach with forward selection. Preliminary models were constructed by sequentially adding eligible variables based on the ascending order of their p-values from the univariable analysis. At each step, we compared the model with and without the new candidate variable using the likelihood ratio test, with a significance threshold set at *p* < 0.05. The biomarkers were included in the multivariable logistic regression model by default, based on the objectives of the study.

Statistical analyses were performed using Stata v. 16.1.

## Results

A total of 279 patients (153 males, median age 76.7 years) were included in the analysis. Demographic, clinical, and laboratory data for the total sample, as well as for the COVID-w/o Neuro and Neuro-COVID groups, are presented in Table 1. Serum NfL levels were assessed in 276 patients, whereas serum S100B was measured in 268 patients. The median time from admission to sample collection was 3 days.

**Table 1:** Characteristics of the study cohort

	Total sample *N* = 279	COVID-w/o Neuro *N* = 210	Neuro-COVID *N* = 69	*p*
Age Median (IQR)	76.7 (59.8–84.4)	77.5 (63.4–84.4)	72.4 (58.2–85)	0.391
Sex, N (%) M	153 (54.8)	107 (51)	46 (66.7)	**0.023**
COVID-19 severity (NIH), N (%) No oxygen therapy (OT) Low-flow OT High-flow OT Mechanical Ventilation	119 (42.7) 108 (38.7) 40 (14.3) 12 (4.3)	88 (41.9) 84 (40) 30 (14.3) 8 (3.8)	31 (44.9) 24 (34.8) 10 (14.5) 4 (5.8)	0.765
NeuroCOVID classification, N (%) Extra-cranial neuro-COVID Mild encephalopathy Severe encephalopathy			9 (13) 36 (52.2) 24 (34.8)	< 0.001
Previous neurological disorders, N (%) Yes	100 (35.8)	64 (30.5)	36 (52.2)	**0.001**
Pulmonary disease, N (%) Yes	57 (20.4)	44 (23)	13 (18.8)	0.706
Smoke, N (%) Yes	66 (23.7)	34 (18.8)	9 (15)	0.261
Obesity, N (%) Yes	51 (18.3)	42 (20)	9 (13)	0.195
Diabetes mellitus, N (%) Yes	82 (29.4)	62 (29.5)	20 (29)	0.932
Ischemic heart disease, N (%) Yes	51 (18.3)	42 (20)	9 (13)	0.195
Hypertension, N (%) Yes	187 (67)	139 (66.2)	48 (69.6)	0.605
Chronic kidney disease, N (%) Yes	62 (22.2)	48 (22.9)	14 (20.3)	0.656
Immunosuppression, N (%) Yes	29 (10.4)	27 (12.9)	2 (2.9)	**0.021**
Fever, N (%) Yes	53 (19)	41 (19.5)	12 (17.4)	0.695
Amines, N (%) Yes	4 (1.4)	2 (1)	2 (2.9)	0.256
Steroids, N (%) Yes	114 (40.9)	94 (44.8)	20 (29)	**0.021**
Tocilizumab, N (%) Yes	2 (0.7)	2 (1)	0 (0)	0.608
sNFL, pg/mL Median (IQR) missing	72.7 (35.5–175.5) 1	68.3 (34.3–153) 0	110 (44.6–244) 1	**0.035**
S100B serum, μg/mL Median (IQR) missing	12 (10.1–13.2) 11	0.13 (0.08–0.23) 1	0.11 (0.08–0.23) 10	0.573
S100B liquor, μg/mL Median (IQR) missing	2.3 (1.8–3.3) 42	-	2.3 (1.8–3.3) 42	-
Q/S100 Median (IQR) missing	0.05 (0.03–1) 43	-	0.05 (0.03–1) 43	-
K-index Median (IQR) missing	1.6 (1.2–3.4) 48	-	1.6 (1.2–3.4) 48	-
Time symptoms-sampling, days Median (IQR)	3 (1–11)	3 (1–12)	2 (1–6)	0.014
White Blood Cells, 10^9^/L Median (IQR) missing	6.6 (4.3–9.3) 1	5.9 (4–8.9) 0	7.3 (5.5–10.2) 1	**0.007**
Lymphocytes, 10^9^/L Median (IQR) missing	1 (0.7–1.5) 27	1 (0.7–1.4) 22	1 (0.8–1.6) 5	0.282
Platelets, 10^9^/L Median (IQR) missing	216.5 (166–281) 1	215.5 (166–280) 0	227.5 (164.5–295.3) 1	0.692
Hemoglobin, g/dL Median (IQR) missing	4.1 (1.1–10.1) 1	11.9 (10–13) 0	12.5 (10.4–13.7) 1	0.129
Na (sodium), mmol/L Median (IQR) missing	139 (136–141) 6	139 (136–141) 3	140 (137–144) 3	0.006
K (potassium), mmol/L Median (IQR) missing	4 (3.7–4.4) 8	4 (3.7–4.4) 5	4.1 (3.7–4.4) 3	0.799
CRP (C-reactive Protein), mg/dL Median (IQR) missing	4.1 (1.1–10.1) 14	4.9 (1.4–11.5) 11	1.9 (0.5–6.1) 3	** < 0.001**
LDH (lactate dehydrogenase), U/L Median (IQR) missing	0.9 (0.7–1.4) 139	235.5 (194–338) 108	276.5 (204–386) 31	0.171
AST (aspartate transaminase), U/L Median (IQR) missing	25 (18–37) 23	24 (18–36) 17	30 (17–45) 6	0.242
Total bilirubin, mg/dL Median (IQR) missing	0.7 (0.5–0.9) 53	0.6 (0.5–0.9) 40	0.7 (0.5–0.9) 13	0.528
CPK (creatine phosphokinase), U/L Median (IQR) missing	75 (34–177) 108	66 (31–146) 94	101 (49–262) 14	**0.009**
Creatinine, mg/dL Median (IQR) missing	3.2 (0.9–535) 10	0.9 (0.7–1.5) 7	1 (0.7–1.4) 3	0.976
PT (prothrombin time) Median (IQR) missing	1.14 (1.08–1.29) 42	1.14 (1.08–1.3) 32	1.13 (1.08–1.25) 10	0.392
ApTT (activated partial thromboplastin time) Median (IQR) missing	1.1 (1–1.4) 54	1.2 (1–1.5) 41	1 (0.9–1.1) 13	< 0.001
D-dimer, mg/L Median (IQR) missing	21 (9.8–48.8) 204	1.1 (0.8–3.1) 164	1,118 (477–1,690) 40	** < 0.001**
Fibrinogen, mg/L Median (IQR) missing	476 (385–579) 148	485 (412–600) 123	434.5 (346) 25	0.014
Ferritin, ng/mL Median (IQR) missing	260 (91–506) 177	266.5 (95–542) 124	177 (59.5–349) 53	0.279
IL-6 (Interleukin 6), pg/mL Median (IQR) missing	2 (0–3) 162	21.3 (9.8–48.8) 120	19.4 (9.8–43.8) 42	0.836
mRs (modified ranking scale) before admission Median (IQR)	1 (0–2)	1 (0–2)	1 (0–2)	0.762
mRs discharge Median (IQR) missing	18 (6.5) 3	1 (0–3) 1	3 (1–4) 2	** < 0.001**
Duration of admission, days Median (IQR) missing	13 (7–25) 6	13 (7–24) 4	16 (7–38) 2	0.142
Deceased during hospitalization, N (%) Yes	18 (6.5)	11 (5.2)	7 (10.3)	0.141

According to COVID severity, 119 patients (42.7%) did not require oxygen therapy, 108 (38.7%) required low-flow oxygen therapy, 40 (14.3%) required high-flow oxygen therapy or non-invasive ventilation and 12 required mechanical ventilation (Table 1).

Sixty-nine (24.7%) patients developed Neuro-COVID and were grouped as follows: 9 (13.0%) had extra-cranial neuro-COVID, 36 (52.2%) had mild encephalopathy and 24 (34.8%) had severe encephalopathy (Table 1).

### Clinical features

In the Neuro-COVID group compared to COVID-w/o Neuro there was a significantly larger proportion of men [46 (66.7%) vs 107 (51%); *p* = 0.023], presence of previous neurological disorders [36 (52.2%) vs 64 (30.5%); *p* = 0.001], and higher mRS at discharge [median values 3 vs 1; *p* < 0.001]; moreover, the Neuro-COVID group presented with a lower proportion of patients with immunosoppression[2 (2.9%) vs 27 (12.9%); *p* = 0.021] and receiving steroids [20 (29%) vs 94 (44.8%); *p* = 0.021] (Table 1).

### Laboratory features

In the Neuro-COVID group compared to COVID-w/o Neuro, there were significantly higher values of NfL [median values 110 vs 68.3 pg/mL; *p* = 0.035], white blood cells [median values 7.3 vs 5.9 10^9^/L; *p* = 0.007], serum sodium [median values 140 vs 139 mmol/L; *p* = 0.006], CPK [median values 101 vs 66 U/L; *p* = 0.009], and D-dimer [median values 1.118 vs 1.1 mg/L; *p* < 0.001], whereas the following were lower: CRP [median values 1.9 vs 4.9 mg/dL; *p* < 0.001], aPTT [median values 1 vs 1.2; *p* < 0.001], and fibrinogen [median values 434.5 vs 485 mg/dL; *p* = 0.014] (Table 1).

### Diagnostic analysis—S100B and NfL biomarkers

The ROC analysis showed very low accuracy in the discrimination between Neuro-COVID vs. COVID-w/o Neuro for NfL and no accuracy for S100B, with an area under the curve (AUC) values respectively of 0.58 (95% CI = 0.51–0.66) and 0.48 (95% CI = 0.39–0.56). The optimal cut-off for NfL, as determined by the Youden Index, was 87.5 pg/mL, with a sensitivity of 57% and specificity of 63%. The optimal cut-off for S100B was 0.134 μg/mL, with a sensitivity of 54% and specificity of 50%.

### Cross sectional analysis

Associations between S100B, NfL and the clinical factors are reported in Table 2.

**Table 2:** Associations between the two biomarkers and other clinical variables

	NFL	*p*	S100B	*p*
Age Rho coefficient	0.296	** < 0.001**	0.250	< 0.001
Sex M F	74.6 (35.9–225) 68.3 (34.9–153)	0.233	0.11 (0.07–0.21) 0.15 (0.09–0.32)	0.004
Previous neurological disorders Yes No	78 (44–240) 69.7 (33.1–164)	**0.049**	0.14 (0.09–0.26) 0.13 (0.07–0.23)	0.099
Pulmonary disease Yes No	85.3 (54–153) 68.7 (32.7–180)	0.063	0.15 (0.10–0.29) 0.11 (0.08–0.22)	0.032
Smoke Yes No	69.1 (37.3–176) 72.7 (33.4–177.5)	**0.019**	0.13 (0.08–0.21) 0.13 (0.08–0.26)	0.476
Diabetes mellitus Yes No	109.5 (44–240) 68.5 (32.1–153)	**0.004**	0.13 (0.09–0.23) 0.13 (0.08–0.23)	0.461
Ischemic heart disease Yes No	116 (56.7–251) 67.8 (32.5–171)	**0.001**	0.13 (0.09–0.25) 0.13 (0.08–0.23)	0.810
Hypertension Yes No	77.3 (45.7–205) 42.5 (12.3–106)	** < 0.001**	0.13 (0.08–0.26) 0.11 (0.06–0.20)	0.042
Chronic kidney disease Yes No	149 (73.9–354) 61.4 (29.8–124)	** < 0.001**	0.16 (0.10–0.28) 0.13 (0.08–0.22)	0.025
Haemoglobin Rho coefficient	−0.509	** < 0.001**	−0.168	0.006
CRP (C-reactive Protein) Rho coefficient	0.110	0.076	0.192	0.002
Creatinine Rho coefficient	0.343	** < 0.001**	0.183	0.003
modified Rankin scale (mRS) before admission Rho coefficient	0.362	** < 0.001**	0.210	< 0.001
NeuroCOVID classification No neurological symptoms Extra-cranial neuro-COVID Mild encephalopathy Severe encephalopathy	68.3 (34.3 – 153) 128 (60.0 – 225.0) 78.4 (34.8 – 187.5) 119 (73.6 – 416)	**0.030**	0.13 (0.08 – 0.23) 0.10 (0.04 – 0.18) 0.11 (0.08 – 0.18) 0.16 (0.08 – 0.33)	0.597
COVID-19 severity (NIH No oxygen therapy (OT) Low-flow OT High-flow OT Mechanical Ventilation	53.8 (27.2–150) 81.8 (43.4–192) 66.4 (37.9–139.5) 117 (91.4–274.5)	**0.005**	0.11 (0.07–0.21) 013 (0.08–0.29) 0.13 (0.10–0.32) 0.14 (0.08–0.23)	0.076

^*^Median sNFL value (IQR) for categorical variables or Spearman correlation for continuous variable

After multivariable regression models the clinical factors positively associated with NfL were age (*β* = 0.02, 95% CI = 0.01–0.03; *p* = 0.001), Chronic Kidney disease (CKD) (*β* = 0.68, 95% CI = 0.34–1.01; *p* < 0.001), extracranial disease vs COVID-w/o Neuro (*β*  = 0.76, 95% CI = 0.12–1.4; *p* = 0.021), severe encephalopathy vs COVID-w/o Neuro (*β* = 0.56, 95% CI = 0.13–0.99; *p* = 0.011), low-flow oxygen therapy vs no O_2_ therapy (*β*  = 0.32, 95% CI = 0.02–0.61; *p* = 0.034), MV vs no O_2_ therapy (*β* = 0.80, 95% CI = 0.19–1.4; *p* = 0.011). The clinical factors found to be inversely associated with NfL were CRP (*β* = −0.03, 95% CI = −0.05/−0.01; *p* = 0.006) and hemoglobin (*β* = −0.22, 95% CI = −0.28/−0.15; *p* < 0.001).

The clinical factor found to be positively associated with S100B was age (*β*  = 0.01, 95% CI = 0.01–0.02; *p* = 0.001), while the clinical factor found to be inversely associated with S100B was Hb (*β*  = −0.06, 95% CI = −0.11/−0.004; *p* = 0.037).

### Prognostic analysis

Associations between worsening and not-worsening of the clinical conditions and biomarkers levels are reported in Table 3

**Table 3:** Characteristics of the study cohort by type of modified Rankin scale (mRS) worsening

	Non-worsening *N* = 192	Worsening *N* = 84	*p*
Age Median (IQR)	76 (60–84.5)	78 (62.1–84.3)	0.752
Sex, N (%) M	108 (71.1)	44 (28.9)	0.552
NeuroCOVID classification, N (%) No neurological symptoms Extra-cranial neuro-COVID Mild encephalopathy Severe encephalopathy	160 (83.3) 1 (0.5) 23 (12) 8 (4.2)	49 (58.3) 7 (8.3) 13 (15.5) 15 (17.9)	< 0.001
COVID-19 severity (NIH), N (%) No oxygen therapy (OT) Low-flow OT High-flow OT Mechanical Ventilation (MV)	90 (46.9) 74 (38.5) 26 (13.5) 2 (1)	28 (33.3) 33 (39.3) 14 (16.7) 9 (10.7)	0.001
Previous neurological disorders, N (%) Yes	75 (39.1)	24 (28.6)	0.095
Pulmonary disease, N (%) Yes	39 (20.3)	18 (21.4)	0.833
Smoke, N (%) Yes	52 (31.5)	14 (19.2)	0.050
Obesity, N (%) Yes	36 (18.8)	14 (16.7)	0.679
Diabetes mellitus, N (%) Yes	59 (30.7)	23 (27.4)	0.575
Ischemic heart disease, N (%) Yes	39 (20.3)	12 (14.3)	0.235
Hypertension, N (%) Yes	127 (66.2)	59 (70.2)	0.505
Chronic kidney disease, N (%) Yes	46 (24)	15 (17.9)	0.261
Immunosuppression, N (%) Yes	23 (12)	6 (7.1)	0.228
Fever, N (%) Yes	37 (19.3)	15 (17.9)	0.782
Amines, N (%) Yes	1 (0.5)	3 (3.6)	0.085
Steroids, N (%) Yes	81 (42.2)	32 (38.1)	0.525
Tocilizumab, N (%) Yes	2 (1)	0 (0)	0.206
sNFL, pg/mL Median (IQR)	65.2 (33.5–138)	110 (47.4–270)	**0.002**
S100B serum, μg/mL Median (IQR)	0.12 (0.08–0.20)	0.15 (0.09–0.34)	0.019
S100B liquor, μg/mL Median (IQR)	1.9 (1.7–2.1)	2.5 (1.8–3.5)	0.100
QS100 Median (IQR)	0.03 (0.03–0.07)	0.06 (0.03–0.1)	0.617
K-index Median (IQR)	1.8 (1.2–3.4)	1.6 (1–2.9)	0.760
Time symptoms-sampling, days Median (IQR)	3 (1–11)	4 (1–8)	0.851
White Blood Cells, 10^9^/L Median (IQR)	5.8 (4–8.8)	7.8 (5.3–10.7)	0.002
Lymphocytes, 10^9^/L Median (IQR)	1 (0.7–1.4)	1.1 (0.7–1.6)	0.392
Platelets, 10^9^/L Median (IQR)	216 (163–281)	221 (171.5–280)	0.999
Haemoglobin, g/dL Median (IQR)	12.1 (10.1–13.2)	11.9 (9.9–13.2)	0.461
Na (sodium), mmol/L Median (IQR)	139 (136–141)	139 (136–141.5)	0.800
K (potassium), mmol/L Median (IQR)	4 (3.7–4.4)	4.1 (3.6–4.5)	0.918
CRP (C-reactive Protein), mg/dL Median (IQR)	4.1 (1.2–9.8)	4.3 (0.8–10.2)	0.758
LDH (lactate dehydrogenase), U/L Median (IQR)	232 (185–326)	298 (224–439)	0.003
AST (aspartate transaminase), U/L Median (IQR)	24 (17–36)	29 (18–37)	0.173
Total bilirubin, mg/dL Median (IQR)	0.7 (0.5–0.9)	0.7 (0.5–0.9)	0.486
CPK (creatine phosphokinase), U/L Median (IQR)	71.5 (30.5–154)	71.5 (30.5–154)	0.666
Creatinine, mg/dL Median (IQR)	0.9 (0.7–1.4)	0.9 (0.7–1.4)	0.548
PT (prothrombin time) Median (IQR)	1.1 (1.1–1.3)	1.1 (1.1–1.3)	0.692
apTT (activated partial thromboplastin time) Median (IQR)	1.2 (1–1.5)	1.1 (1–1.3)	0.031
D-dimer, mg/L Median (IQR)	1.1 (0.7–23.6)	17.7 (1.7–1,351)	< 0.001
Fibrinogen, mg/L Median (IQR)	473 (411–569)	486 (345.5–599)	0.835
Ferritin, ng/mL Median (IQR)	194 (86–496)	268 (190–542)	0.188
IL-6 (Interleukin 6), pg/mL Median (IQR)	20.4 (6.6–48.8)	22.9 (16.2–43.8)	0.103
mRs before admission Median (IQR)	1 (0–3)	1 (0–2)	0.423
mRs discharge Median (IQR)	1 (0–3)	3 (2–4.5)	< 0.001
Duration of admission, days Median (IQR)	34.3 (37.1)	22 (12–46)	< 0.001
Deceased during hospitalization, N (%) Yes	0 (0)	18 (21.4)	< 0.001

After multivariable regression models the variables that contributed to worsening of mRS were MV vs no O_2_ therapy (OR = 9.56, 95% CI = 1.67–54.75; *p* = 0.011), severe encephalopathy vs COVID-w/o Neuro (OR = 5.10, 95% CI = 1.58–16.19; *p* = 0.006), S100B (per 1-μg/mL increase OR = 2.62, 95% CI = 1.10–6.46; *p* = 0.037) and duration of admission (per 1-day increase OR = 1.03, 95% CI = 1.01–1.05; *p* = 0.001). Predictors of worsening after multivariable regression analysis are reported in Table 4.

**Table 4:** Predictor of modified Rankin scale (mRS) worsening after multivariable logistic regression

Variables	Odds Ratio	95% CI	*p*-value
COVID-19 severity (NIH), N (%) No oxygen therapy (OT) vs Low-flow OT High-flow OT Mechanical Ventilation	1.21 1.33 **9.55**	0.59 – 2.48 0.52 – 3.39 **1.67 – 54.75**	0.611 0.547 **0.011**
NeuroCOVID classification, N (%) No neurological symptoms vs Extra-cranial neuro-COVID Mild encephalopathy Severe encephalopathy	10.16 1.91 **5.06**	0.89 – 115.92 0.77 – 4.70 **1.58 – 16.19**	0.062 0.159 **0.006**
sNFL – 1 pg/ml increase	0.999	0.999 – 1.002	0.753
S100B_serum – 1 μg/mL increase	**2.62**	**1.06 – 6.46**	**0.037**
Duration of admission – 1 day increase	**1.03**	**1.01 – 1.05**	**0.001**
mRs before admission – 1 point increase	0.84	0.66 – 1.09	0.195

## Discussion

In this multicenter study involving a large cohort of COVID-19 patients, serum NfL and S100B biomarkers were not accurate in discriminating cases with neurological manifestations from those without. However, S100B displayed a prognostic value, as the elevated levels of this biomarker predicted in our cohort a functional worsening. Besides, our results are in agreement with findings of no utility of S100B in determining COVID-19 acute severity, meant as necessity of respiratory support according to NIH classification [19].

Nonetheless, NfL displayed a positive correlation with severe encephalopathy and extracranial neurological manifestations, compared to the population which did not develop any neurological symptoms. No direct relation was observed in patients with mild encephalopathy. These findings suggest that NfL might be less sensitive for mild conditions, and that finding a high level of NfL in patients with COVID-19 should be considered as a potential biomarker of severe neurological involvement and the need for tempestive treatment. The fact the NfL might not be able to effectively discriminate neuro-COVID patients might stem from the presence of neuroaxonal injury also in mild COVID-19 patients and those without neurological manifestations [23].

The prediction of neurological complications is a fundamental tool in the management of patients with high-severity COVID-19, as it has been observed that the development of CNS involvement can severely impact patient outcome [28]. In particular, patients admitted to the hospital with only respiratory symptoms but who developed neurological conditions during the hospitalization are reported to progress more frequently with a more severe course, with increased intubation and mechanical ventilation requirements, although it is more frequent in patients presenting with more compromised respiratory function [24]. Accordingly, in addition to S100B, the functional prognosis in our cohort was impacted by the occurrence of MV and the presence of severe encephalopathy.

Our results are also in agreement with early pandemic findings of the prognostic role of elevated D-dimer levels, especially when higher than 1 μg/mL, in patients with poor outcome [29]. D-dimer is a marker of increased coagulation activity, determined by a systemic pro-inflammatory cytokine response, which can contribute to plaque disrupture predisposing to ischemia and thrombosis. Also, the presence of ACE2, the receptor for SARS-CoV-2, in the vascular endothelial cells, can contribute to the mechanism [29].

The mechanisms underlining the central and peripheral nervous system involvement during the infection are still not clearly defined, although it is evident that multi-factorial damage of the BBB can occur and explain the clinical manifestations. In particular, it has been shown that SARS-CoV-2 may determine a state of hypercoagulability and hyperinflammation that may lead to the disruption of the BBB, also by interacting with the ACE2 receptors in the capillary walls of the brain circulation [30].

The possible role in the onset of neurological symptoms of systemic inflammation and BBB disruption has been investigated through the correlation of blood biomarkers of BBB disruption, neuronal damage (NfL), and systemic inflammation and clinical findings [31]. Specifically, it has been observed that blood biomarkers of BBB disruption and neuronal damage are high in COVID-19 patients, with levels similar to or higher than in patients with other neurological illnesses (i.e. ALS) [31]. Higher levels of NfL in patients with severe COVID and neurological manifestations were previously observed and are in line with our findings [31].

Additionally, we found that neuro-COVID was negatively correlated with immunosuppression and steroid usage is noteworthy and could be interpreted in various ways. On one hand, reduced steroid therapy might have resulted in decreased neuroprotection, subsequently leading to the development of neurological symptoms. On the other hand, neuro-COVID patients may have required less intensive systemic treatment. Nevertheless, the severity of acute respiratory COVID-19 was similar between the two groups.

We have also observed a relation between high levels of both NfL and S100B and low hemoglobin, which we considered as a sign of systemic inflammation, as hemoglobin tends to drop in the presence of activation of the inflammatory system.

The impact of our research is that we caution other researchers not to overinterpred results obtained in critical ill patients displaying several features affecting the fate of these biomarkers. Notably, we found high levels of the biomarkers in patients with reduced or absent neurological manifestations, suggesting potential confounding factors such as reduced kidney filtration impacting biomarker levels.

The study presents some limitations which may have influenced the investigated associations. First, the control group (i.e., non-neuro-COVID) was not visited by a neurologist, determining a possibile underestimation of neurological symptoms in our cohort. Especially for patients with severe disease, it is possible that concomitant nervous damage was present and not captured, acting as a confounder impacting the specificity of both biomarkers. Secondarily, not all data were available for the entire population, potentially leading to an overestimation or underestimation of the relevance of some parameters. Additionally, the mRS was used for the prognostic analysis, although this disability score is designed for neurological conditions and is usually evaluated at follow-up evaluations, not included in the study design due to healthcare challenges during the pandemic. As no gold-standard classification of neuro-COVID symptoms exists, we classified them arbitrarily into three categories: although these may not capture perfectly the whole spectrum of neurological manifestations, these allow the discrimination between milder and more severe cases. Finally, there was not a strict timing in sampling, therefore some values may have been sampled at different stages of their evolutionary trend.

## Conclusions

We found that blood S100B nor NfL biomarkers were able to discriminate between patients with and without neurological manifestations.. While S100B was not associated with neuro-COVID, in alignment with previous findings, it showed prognostic value by predicting poor outcomes in COVID patients. On the other hand, serum NfL was associated with extracranial neurological manifestations and severe encephalopathy, corroborating its potential as a biomarker of severe neurological conditions. Our study raises important considerations regarding the interpretation of biomarker results in critically ill patients with multiple comorbidities. We suggest caution against overinterpreting biomarker data in these complex conditions, emphasizing the need to account for factors such as renal function and systemic inflammation.

## Data Availability

The raw data that support the findings of this study are available upon reasonable request.
